# *catena*-Poly[[(pyridine-κ*N*)copper(II)]-μ_3_-pyridine-2,6-dicarboxylato-κ^3^ *O* ^2^:*O* ^2′^,*N*,*O* ^6^:*O* ^6′^]

**DOI:** 10.1107/S1600536809005212

**Published:** 2009-02-21

**Authors:** Manoj Trivedi, Daya Shankar Pandey, Nigam P. Rath

**Affiliations:** a Department of Chemistry, Faculty of Science, Banaras Hindu University, Varanasi 221005, India; b Department of Chemistry and Biochemistry, and Centre for Nanoscience, University of Missouri-St Louis, One University Boulevard, St Louis, MO 63121-4499, USA

## Abstract

In the title compound, [Cu(C_7_H_3_NO_4_)(C_5_H_5_N)]_*n*_, the Cu^II^ atom is in a slightly distorted octa­hedral coordination environment. Each Cu^II^ atom is bound to two N atoms and one O atom of the pyridine­dicarboxyl­ate (PDA) ligand in a tridentate manner, one N atom of the pyridine mol­ecule and two bridging carboxyl­ate O atoms of adjacent PDA ligands, leading to a linear one-dimensional chain running along the *c* axis. These chains are further assembled *via* weak C—H⋯O and π–π inter­actions into a three-dimensional supra­molecular network structure. The centroid–centroid distance between the π–π inter­acting pyridine rings is 3.9104 (13) Å. The two N atoms are *trans* to each other with respect to Cu.

## Related literature

For background information on coordination polymers, see: Kitagawa *et al.* (2004 ▶); Kirillov *et al.* (2008 ▶); Hoskins & Robson (1990 ▶); Eddaoudi *et al.* (2001 ▶). For related polymeric structures of PDA complexes, see, for example: Zhao *et al.* (2003 ▶); Choi *et al.* (2003 ▶); Ghosh *et al.* (2004 ▶); Xie *et al.* (2004 ▶). For related structures of Cu complexes, see: Uçar *et al.* (2007 ▶); Manna *et al.* (2007 ▶); Gao *et al.* (2006 ▶).
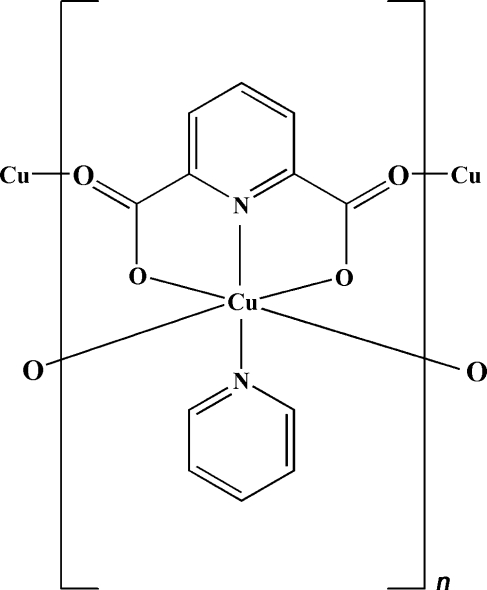


## Experimental

### 

#### Crystal data

[Cu(C_7_H_3_NO_4_)(C_5_H_5_N)]
*M* *_r_* = 307.74
Monoclinic, 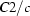

*a* = 7.8042 (9) Å
*b* = 13.6152 (17) Å
*c* = 10.0667 (12) Å
β = 91.687 (4)°
*V* = 1069.2 (2) Å^3^
*Z* = 4
Mo *K*α radiation
μ = 2.06 mm^−1^
*T* = 100 K
0.21 × 0.13 × 0.08 mm

#### Data collection

Bruker APEXII CCD area-detector diffractometer
Absorption correction: multi-scan (**SADABS**; Bruker, 2007 ▶) *T*_min_ = 0.671, *T*_max_ = 0.848
3530 measured reflections
981 independent reflections
859 reflections with *I* > 2σ(*I*)
*R*_int_ = 0.036

#### Refinement

*R*[*F* ^2^ > 2σ(*F* ^2^)] = 0.029
*wR*(*F* ^2^) = 0.074
*S* = 1.10
981 reflections
89 parameters
H-atom parameters not refined
Δρ_max_ = 0.41 e Å^−3^
Δρ_min_ = −0.60 e Å^−3^

## Supplementary Material

Crystal structure: contains datablocks I, global. DOI: 10.1107/S1600536809005212/is2384sup1.cif

Structure factors: contains datablocks I. DOI: 10.1107/S1600536809005212/is2384Isup2.hkl

Additional supplementary materials:  crystallographic information; 3D view; checkCIF report

## Figures and Tables

**Table 1:** Selected bond lengths (Å)

Cu1—N2	1.896 (3)
Cu1—N1	1.944 (3)
Cu1—O1	2.0110 (18)
Cu1—O1^i^	2.0110 (18)

Symmetry code: (i) 
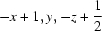
.

**Table 2:** Hydrogen-bond geometry (Å, °)

*D*—H⋯*A*	*D*—H	H⋯*A*	*D*⋯*A*	*D*—H⋯*A*
C3—H3⋯O2^ii^	0.95	2.44	3.187 (3)	135
C5—H5⋯O1^i^	0.95	2.48	3.070 (3)	120
C6—H6⋯O1^iii^	0.95	2.48	3.394 (3)	162

Symmetry codes: (i) 
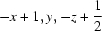
; (ii) 
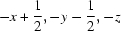
; (iii) 
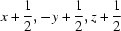
.

**Fig. 1.**
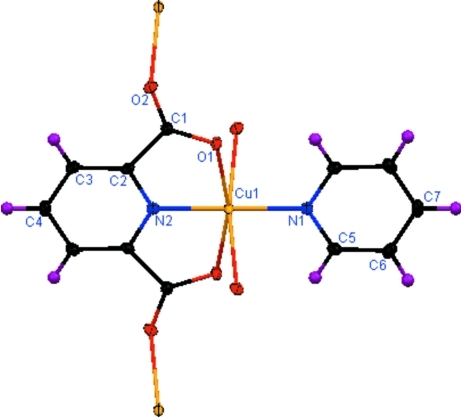
A view of the structure of (1), showing the atom-numbering scheme and the Cu coordination octahedra; displacement ellipsoids are drawn at the 50% probability level.

**Fig. 2.**
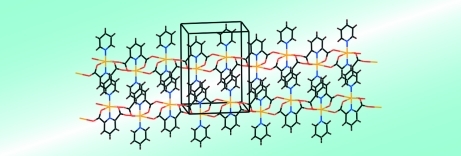
Packing view of (1), showing linear chains along the [001] direction.

**Fig. 3.**
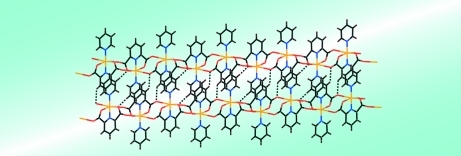
Packing view of (1), showing connectivity with other polymeric chains through weak C—H···O hydrogen bond interactions.

**Fig. 4.**
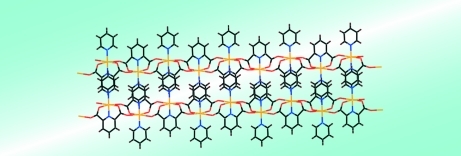
Packing view of (1), showing face-to-face π-π interactions.

## supplementary crystallographic information

### Comment

The rapidly expanding field of the crystal engineering (the design of crystalline materials) of polymeric coordination networks stems has recently attracted great interest because of their potential applications as zeolite-like materials for molecular selection, ion exchange, and catalysis, as well as in the variety of architectures and topologies (Kitagawa *et al*., 2004; Kirillov *et al.*, 2008). The main strategy popularly used in this area is a building-block approach (Hoskins & Robson, 1990; Eddaoudi *et al.*, 2001). 2,6-Pyridinedicarboxylic acid (H_2_PDA) is an efficient ligand. Polymeric structure of PDA complexes with transition and lanthanide metals have been reported, in which PDA not only chelates but also bridges to form diversified structures with three coordination sites (Zhao *et al.*, 2003; Choi *et al.*, 2003; Ghosh *et al.*, 2004; Xie *et al.*, 2004). We report the synthesis, and crystal structures of one compound, [Cu(*µ*-2,6-PDA)(py)]*_n_*, (1).

Molecular structure of (1) shows a slightly distorted octahedral coordination geometry. The equatorial sites are occupied by an NO_2_ donor from the carboxylate groups at the pyridine-2,6-position of PDA (N2, O1, O1^i^) and one N atom from pyridine (N1). Two O atoms from two other neighboring PDA ligands occupy the axial sites (O2, O2^i^) at a distance of 2.761 Å (Fig. 1). The equatorial Cu—O and Cu—N bond lengths of are normal [Cu1—O1 = 2.0110 (18) Å, Cu1—O1^i^ = 2.0110 (18) Å, Cu1—N2 = 1.896 (3) Å, Cu1—N1 = 1.944 (3) Å], which are within ranges reported in other copper complexes (Uçar *et al.*, 2007; Manna *et al.*, 2007; Gao *et al.*, 2006). The pyridine is essentially planar with no deviation from planarity for pyridyl N1-atom. The C—C—C angles about the pyridyl ring are 118.2 (3) to 128.5 (2)°, indicating *sp*^2^ hybridization. Two carboxylate O atoms (O2 and O2^i^) which are coordinated to the adjacent copper atom, have C—O distances [O2—C1 = 1.228 (3) Å, O1—C1 = 1.290 (3) Å] which are generally shorter than C—O distances, indicating the conjugation of the double bond after deprotonation. In this way, the PDA ligands bridge adjacent Cu atoms to form a [Cu(*µ*-2,6-PDC)(py)]*n* linear chains extending in the [001] direction (Fig. 2). The separations between the two Cu atom in the linear chains are 5.332 Å. The PDA ligand and pyridine are *trans* to each other (N2—Cu1—N1 = 180°). However, one-dimensional polymeric chains are connected in the solid state through weak C—H···O and π-π interactions. Weak C—H···O interactions that connects polymeric chains into two-dimensional network (Fig. 3). Contact distances for C—H···O interactions are 2.43–2.76 Å (Table 1). The weak π-π interactions are present in (1). Further, the importance of π-π stacking interactions between aromatic rings has widely been recognized in the intercalation of drugs with DNA especially in biological systems, which lie in the range 3.4–3.5 Å. The complex (1) exhibits intermolecular face-to-face π-π interactions [π-pyridyl/π-pyridyl ct/ct distance 3.9104 (13) Å; Fig. 4].

### Experimental

A mixture of [Cu(NO_3_)_2_.6H_2_O] (0.466 g, 2 mmol), H_2_PDA (0.167 g, 1 mmol), 2-Pyridine thiol(0.111 g, 1 mmol) was dissolved in a mixture of MeOH (5 ml) and water (5 mL) and add pyridine (in excess). The solution was stirred for 24 h at room temperature. Slowly, color of the solution changes from blue to dark green. The resulting solution was filtered and left at room temperature for two days, which resulted in blue needle crystals which are suitable for X-ray diffraction analysis (yield 0.184 g, 60%). Anal. Calc. for C_12_H_8_N_2_O_4_Cu: C 48.83, H 2.62, N 9.10%; found: C 47.65, H 2.56, N 9.30%.

### Refinement

All H atoms were added in their calculated positions (C—H = 0.95 Å) and were treated using appropriate riding models, with *U*_iso_(H) = 1.2*U*_eq_(C).

### Crystal data

**Table:**

[Cu(C_7_H_3_NO_4_)(C_5_H_5_N)]	*F*(000) = 620
*M**_r_* = 307.74	*D*_x_ = 1.912 Mg m^−^^3^
Monoclinic, *C*2/*c*	Mo *K*α radiation, λ = 0.71073 Å
Hall symbol: -C 2yc	Cell parameters from 1607 reflections
*a* = 7.8042 (9) Å	θ = 3.0–25.3°
*b* = 13.6152 (17) Å	µ = 2.06 mm^−^^1^
*c* = 10.0667 (12) Å	*T* = 100 K
β = 91.687 (4)°	Needle, blue
*V* = 1069.2 (2) Å^3^	0.21 × 0.13 × 0.08 mm
*Z* = 4	

### Data collection

**Table:**

Bruker APEXII CCD area-detector diffractometer	981 independent reflections
Radiation source: fine-focus sealed tube	859 reflections with *I* > 2σ(*I*)
graphite	*R*_int_ = 0.036
φ and ω scans	θ_max_ = 25.3°, θ_min_ = 3.0°
Absorption correction: multi-scan (*SADABS*; Bruker, 2007)	*h* = −9→8
*T*_min_ = 0.671, *T*_max_ = 0.848	*k* = −16→16
3530 measured reflections	*l* = −12→11

### Refinement

**Table:**

Refinement on *F*^2^	Primary atom site location: structure-invariant direct methods
Least-squares matrix: full	Secondary atom site location: difference Fourier map
*R*[*F*^2^ > 2σ(*F*^2^)] = 0.029	Hydrogen site location: inferred from neighbouring sites
*wR*(*F*^2^) = 0.074	H-atom parameters not refined
*S* = 1.10	*w* = 1/[σ^2^(*F*_o_^2^) + (0.0347*P*)^2^ + 1.5139*P*] where *P* = (*F*_o_^2^ + 2*F*_c_^2^)/3
981 reflections	(Δ/σ)_max_ < 0.001
89 parameters	Δρ_max_ = 0.41 e Å^−^^3^
0 restraints	Δρ_min_ = −0.60 e Å^−^^3^

### Special details

**Table:**

Experimental. All H atoms were added in their calculated positions and were treated using appropriate riding models.
Geometry. All e.s.d.'s (except the e.s.d. in the dihedral angle between two l.s. planes) are estimated using the full covariance matrix. The cell e.s.d.'s are taken into account individually in the estimation of e.s.d.'s in distances, angles and torsion angles; correlations between e.s.d.'s in cell parameters are only used when they are defined by crystal symmetry. An approximate (isotropic) treatment of cell e.s.d.'s is used for estimating e.s.d.'s involving l.s. planes.
Refinement. Refinement of *F*^2^ against ALL reflections. The weighted *R*-factor *wR* and goodness of fit *S* are based on *F*^2^, conventional *R*-factors *R* are based on *F*, with *F* set to zero for negative *F*^2^. The threshold expression of *F*^2^ > σ(*F*^2^) is used only for calculating *R*-factors(gt) *etc*. and is not relevant to the choice of reflections for refinement. *R*-factors based on *F*^2^ are statistically about twice as large as those based on *F*, and *R*- factors based on ALL data will be even larger.

### Fractional atomic coordinates and isotropic or equivalent isotropic displacement parameters (Å^2^)

**Table:**

	*x*	*y*	*z*	*U*_iso_*/*U*_eq_	
Cu1	0.5000	0.06463 (3)	0.2500	0.01055 (18)	
O1	0.3461 (2)	0.04164 (13)	0.08928 (18)	0.0119 (4)	
O2	0.2652 (2)	−0.07906 (13)	−0.05100 (19)	0.0137 (4)	
N1	0.5000	0.2074 (2)	0.2500	0.0099 (7)	
N2	0.5000	−0.0746 (2)	0.2500	0.0096 (7)	
C1	0.3334 (3)	−0.04888 (19)	0.0526 (3)	0.0111 (6)	
C2	0.4161 (3)	−0.1210 (2)	0.1514 (3)	0.0100 (6)	
C3	0.4135 (3)	−0.2221 (2)	0.1484 (3)	0.0120 (6)	
H3	0.3542	−0.2564	0.0789	0.014*	
C4	0.5000	−0.2728 (3)	0.2500	0.0130 (8)	
H4	0.5000	−0.3426	0.2500	0.016*	
C5	0.5748 (3)	0.2583 (2)	0.3507 (3)	0.0124 (6)	
H5	0.6286	0.2230	0.4218	0.015*	
C6	0.5765 (4)	0.3594 (2)	0.3549 (3)	0.0154 (6)	
H6	0.6290	0.3931	0.4281	0.018*	
C7	0.5000	0.4112 (3)	0.2500	0.0159 (9)	
H7	0.5000	0.4810	0.2500	0.019*	

### Atomic displacement parameters (Å^2^)

**Table:**

	*U*^11^	*U*^22^	*U*^33^	*U*^12^	*U*^13^	*U*^23^
Cu1	0.0144 (3)	0.0059 (3)	0.0109 (3)	0.000	−0.00649 (18)	0.000
O1	0.0151 (10)	0.0074 (10)	0.0129 (10)	−0.0014 (8)	−0.0066 (8)	0.0000 (8)
O2	0.0173 (10)	0.0117 (11)	0.0118 (10)	−0.0026 (8)	−0.0068 (8)	−0.0017 (8)
N1	0.0095 (16)	0.0095 (17)	0.0108 (17)	0.000	−0.0009 (13)	0.000
N2	0.0085 (16)	0.0102 (17)	0.0100 (16)	0.000	−0.0009 (13)	0.000
C1	0.0114 (14)	0.0106 (14)	0.0114 (15)	−0.0017 (11)	−0.0007 (11)	0.0004 (11)
C2	0.0080 (13)	0.0125 (15)	0.0095 (14)	−0.0020 (11)	−0.0011 (11)	−0.0023 (11)
C3	0.0122 (14)	0.0131 (15)	0.0108 (14)	−0.0006 (11)	−0.0019 (11)	−0.0018 (11)
C4	0.014 (2)	0.009 (2)	0.016 (2)	0.000	0.0001 (16)	0.000
C5	0.0124 (14)	0.0138 (15)	0.0110 (15)	0.0001 (11)	−0.0012 (11)	0.0007 (11)
C6	0.0163 (15)	0.0136 (15)	0.0163 (16)	−0.0035 (12)	0.0025 (12)	−0.0042 (12)
C7	0.016 (2)	0.009 (2)	0.023 (2)	0.000	0.0055 (17)	0.000

### Geometric parameters (Å, °)

**Table:**

Cu1—N2	1.896 (3)	C2—C3	1.378 (4)
Cu1—N1	1.944 (3)	C3—C4	1.392 (3)
Cu1—O1	2.0110 (18)	C3—H3	0.9500
Cu1—O1^i^	2.0110 (18)	C4—C3^i^	1.392 (3)
O1—C1	1.290 (3)	C4—H4	0.9500
O2—C1	1.228 (3)	C5—C6	1.378 (4)
N1—C5^i^	1.347 (3)	C5—H5	0.9500
N1—C5	1.347 (3)	C6—C7	1.390 (3)
N2—C2	1.332 (3)	C6—H6	0.9500
N2—C2^i^	1.332 (3)	C7—C6^i^	1.390 (3)
C1—C2	1.527 (4)	C7—H7	0.9500
			
N2—Cu1—N1	180.0	N2—C2—C1	111.7 (2)
N2—Cu1—O1	81.05 (5)	C3—C2—C1	128.5 (2)
N1—Cu1—O1	98.95 (5)	C2—C3—C4	118.2 (3)
N2—Cu1—O1^i^	81.05 (5)	C2—C3—H3	120.9
N1—Cu1—O1^i^	98.95 (5)	C4—C3—H3	120.9
O1—Cu1—O1^i^	162.10 (10)	C3—C4—C3^i^	120.6 (4)
C1—O1—Cu1	114.71 (16)	C3—C4—H4	119.7
C5^i^—N1—C5	118.1 (3)	C3^i^—C4—H4	119.7
C5^i^—N1—Cu1	120.97 (16)	N1—C5—C6	122.8 (3)
C5—N1—Cu1	120.96 (16)	N1—C5—H5	118.6
C2—N2—C2^i^	123.4 (3)	C6—C5—H5	118.6
C2—N2—Cu1	118.28 (16)	C5—C6—C7	118.7 (3)
C2^i^—N2—Cu1	118.28 (16)	C5—C6—H6	120.7
O2—C1—O1	126.2 (2)	C7—C6—H6	120.7
O2—C1—C2	120.2 (2)	C6^i^—C7—C6	119.0 (4)
O1—C1—C2	113.6 (2)	C6^i^—C7—H7	120.5
N2—C2—C3	119.8 (3)	C6—C7—H7	120.5
			
N2—Cu1—O1—C1	−6.98 (18)	Cu1—N2—C2—C3	−179.96 (18)
N1—Cu1—O1—C1	173.02 (18)	C2^i^—N2—C2—C1	−179.7 (2)
O1^i^—Cu1—O1—C1	−6.98 (18)	Cu1—N2—C2—C1	0.3 (2)
O1—Cu1—N1—C5^i^	−6.84 (14)	O2—C1—C2—N2	172.9 (2)
O1^i^—Cu1—N1—C5^i^	173.16 (14)	O1—C1—C2—N2	−6.2 (3)
O1—Cu1—N1—C5	173.16 (14)	O2—C1—C2—C3	−6.8 (4)
O1^i^—Cu1—N1—C5	−6.84 (14)	O1—C1—C2—C3	174.1 (3)
O1—Cu1—N2—C2	3.29 (14)	N2—C2—C3—C4	−0.1 (4)
O1^i^—Cu1—N2—C2	−176.71 (14)	C1—C2—C3—C4	179.6 (2)
O1—Cu1—N2—C2^i^	−176.71 (14)	C2—C3—C4—C3^i^	0.03 (18)
O1^i^—Cu1—N2—C2^i^	3.29 (14)	C5^i^—N1—C5—C6	0.47 (19)
Cu1—O1—C1—O2	−170.2 (2)	Cu1—N1—C5—C6	−179.53 (19)
Cu1—O1—C1—C2	8.8 (3)	N1—C5—C6—C7	−0.9 (4)
C2^i^—N2—C2—C3	0.04 (18)	C5—C6—C7—C6^i^	0.44 (18)

Symmetry codes: (i) −*x*+1, *y*, −*z*+1/2.

### Hydrogen-bond geometry (Å, °)

**Table:**

*D*—H···*A*	*D*—H	H···*A*	*D*···*A*	*D*—H···*A*
C3—H3···O2^ii^	0.95	2.44	3.187 (3)	135
C5—H5···O1^i^	0.95	2.48	3.070 (3)	120
C6—H6···O1^iii^	0.95	2.48	3.394 (3)	162

Symmetry codes: (ii) −*x*+1/2, −*y*−1/2, −*z*; (i) −*x*+1, *y*, −*z*+1/2; (iii) *x*+1/2, −*y*+1/2, *z*+1/2.
